# Oculopharyngeal muscular dystrophy as a rare differential diagnosis for unexplained dysphagia: a case report

**DOI:** 10.1186/1757-1626-2-94

**Published:** 2009-01-28

**Authors:** Klaus Bumm, Martin Zenker, Alessandro Bozzato

**Affiliations:** 1 Department of Otorhinolaryngology-Head and Neck Surgery, University of Erlangen-Nuremberg, Germany; 2 Institute of Human Genetics, University of Erlangen-Nuremberg, Germany

## Abstract

**Introduction:**

We wish to report on a rare cause of dysphagia; oculopharyngeal muscular dystrophy (OPMD). It is a late adult onset autosomal dominant form of muscular dystrophy that constitutes as a rare diagnosis for any place outside of Canada and first case in southern Germany.

**Case presentation:**

We report the medical odyssey of a 57-year old male Caucasian patient. He was referred at our hospital for further clarification of a progressive dysphagia, which, at first view, was thought to be tumor related due to the patient's typical anamnesis.

**Conclusion:**

The present report outlines the importance of considering this rare disease for general medicine practitioners as well as head and neck specialists as a differential diagnosis for swallowing disorders with, even at second view, uncertain cause.

## Case presentation

A 57-year old Caucasian male patient with acute pneumonia and a progressive dysphagia was referred to our department. He reported episodes of painless swallowing difficulties, associated with xerostomia, hoarseness and an uncomfortable sensation of a mass in the upper esophagus. His general medical condition includes type II diabetes, chronic bronchitis, chronic gastritis, urate nephropathy with a history of nephrolithiasis and coronary artery disease. The patient reported daily alcohol and nicotine abuse over a period of 40 years.

**Figure 1:**
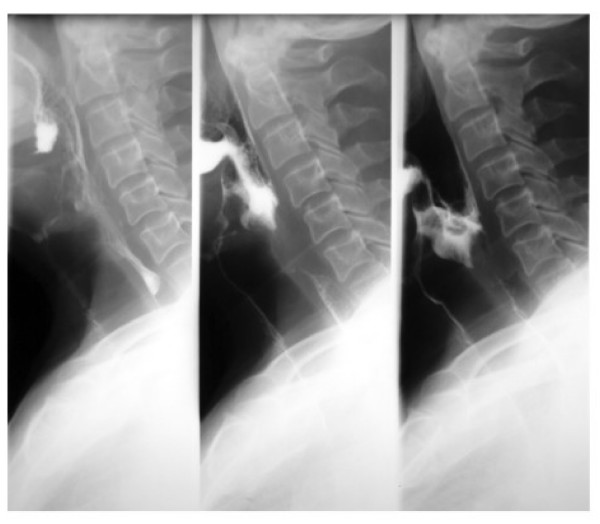
**Video-radiological barium examination with intra- and- postdeglutive aspiration**. From left to right: Oral phase of the swallowing procedure (left picture), laryngeal phase of the swallowing procedure (middle picture) and prolonged laryngeal/pharyngeal phase (right picture) with aspiration into the larynx and trachea.

On physical examination he showed a distinct bilateral eyelid ptosis combined with a mild facial weakness. Wet voice and an insufficient cough reflex after solid and liquid intake followed by consecutive aspiration were observed. Laryngoscopy showed an incomplete glottic closure and impaired vocal cord mobility. Video-radiological barium examination revealed intra- and- postdeglutive aspiration (Figure 1). Contrast enhanced CT scans showed an unclear homogeneous mass (1.5 × 2 cm) located in the preepiglottic space, dorsal to the hyoid bone.

We performed a panendoscopy under general anesthesia and esophagus-gastroscopy, suspecting a malignant process. Endoscopic findings neither confirmed a tumorous process nor revealed any other obvious cause for the progressive dysphagia. Therefore a deep incision biopsy of the preepiglottic space was performed, but no suspect structures other than fat tissue were noted. Histopathological evaluation of the preepiglottic mass confirmed solely fatty tissue without malignant pathology.

**Figure 2:**
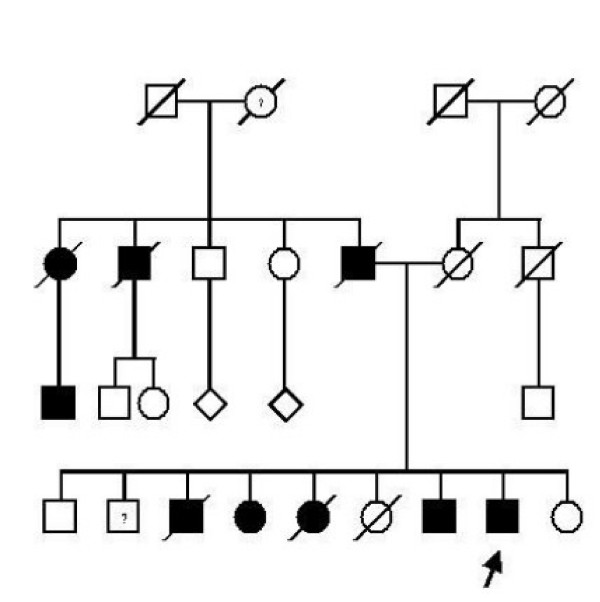
**Pedigree of the patient (marked by arrow) indicating autosomal-dominant transmission of OPMD**. One 69 year old brother (marked by a question mark) of the index patient was reported to have only mild dysphagia without eyelid ptosis which may reflect mild expression of the disease or even chance association. The affection status of the paternal grandmother (marked by a question mark) is unclear because she died already at the age of 36 years.

Family history revealed that he has eight siblings, four of which developed similar symptoms at around 50 years of age. One brother has only mild dysphagia. Furthermore the patient's father, one paternal uncle, one paternal aunt and her son suffer from dysphagia and eyelid ptosis. (Figure 2) The patient's family is native German without French-Canadian or Jewish roots. The patient underwent bilateral blepharoplasty surgery to correct eyelid ptosis one year ago.

**Figure 3:**
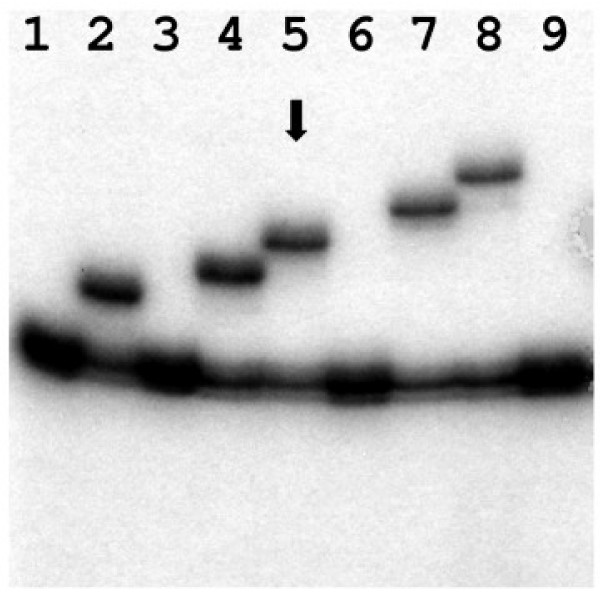
**Analysis of GCG-repeat size in exon 1 of the PABPN1 gene by PCR and denaturing PAA gel electrophoresis as described previously showing heterozygosity for a (GCG)10 expansion in the index patient (lane 5, arrow)**. Lanes 1, 3, 6, and 9 represent normal homozygous controls for the (GCG)6 allele. Lanes 2, 4, 7 and 8 show heterozygote and non related carriers of (GCG)8, (GCG)9, (GCG)11, and (GCG)12 expansions, respectively.

Considering the family history of swallowing disorders, we suspected a hereditary cause of the dysphagia and initiated genetic counseling. DNA testing revealed a heterozygous (GCG)10 expansion of the (GCG)-mini-repeat, located in exon 1 of the poly(A) binding protein, nuclear 1gene (PABPN1), thus verifying oculopharyngeal muscular dystrophy (Figure 3).

After the diagnosis was confirmed and therapeutic options were discussed, the patient refused a cricopharyngeal myotomy. Therefore a percutaneous endoscopic gastrostomy (PEG) placement was carried out to improve the overall nutritional situation. After a period of five months, he reported on unsteady functional improvements of his swallowing. Other neurological functions remained unchanged. The PEG probe was left in place since sufficient oral deglutition did not occur.

## Discussion and conclusion

Patients suffering from multiple ailments are common in ENT hospitals and many exhibit dysphagia at some point. Especially in patients with long periods of alcohol and nicotine abuse, dysphagia is mostly related to malignant processes of the hypopharynx, larynx or esophagus. This patients underwent a series of hospitalizations simply because physicians would not think of a disease as rare as OPMD as a cause for dysphagia in Germany.

Most cases and families suffering from OPMD are located in Canada [1] among the Bukhara Jewish community [2] originating from Uzbekistan. For any place other than Canada, where the largest cluster of OPMD cases is found in the Province of Quebec (incidence 1:1000), this disease is uncommon and almost always unfamiliar to head and neck specialists or neurologists. OPMD was first described in 1915 and in 1962 it was referred to as OPMD. Recent studies were able to show that OPMD cases in Canada can be traced back to a small group of French immigrants [3]. To date OPMD has been reported in over 20 countries with an incidence so low; it has not yet been appraised [3,4].

OPMD usually manifests in the 4^th ^to 6^th ^decade with slowly progressive eyelid ptosis and dysphagia due to impaired levator palpebrae and pharyngeal muscle function. In most cases the eyelid ptosis is the first symptom and is almost always closely followed by a progressive dysphagia. At a later stage and as the disease evolves, extraocular and facial muscles may also become affected. However, eyelid ptosis is generally recognized as the first and cardinal symptom. In a minor percentage, patients develop weakness and atrophy of the tongue, pelvic girdle muscles and to a lesser extent the shoulder girdle muscles [5].

The diagnosis can only be finalized by DNA testing of CGC triplets. To date there is no causal treatment for this disease. A high-protein diet is recommended for optimal nutritional balance. Aspiration pneumonia, as in the reported case, is a frequent complication; therefore, early medical supervision should be sought. At this time, only surgical treatments to correct the functional aspects of OPMD is available [6]. Surgical correction of the ptosis and a cricopharyngeal myotomy has been reported as effective surgical treatment. Owing to symptomatic treatment and alternative nutritional options, life expectancy is not reduced by the condition. Data regarding the survival of untreated OPMD does not exist. Outside of Canada, OPMD reports from neurologists are rare; from otolaryngologists they are scarce and they have so far not been made from general practitioners. In a time where mobility and relocation is facilitated by air travel, exotic diagnoses increase. Therefore it is important to have OPMD in mind when often dealing with a large group of patients suffering from dysphagia, as most head and neck specialists do. Due to the rather unspecific nature of the cardinal symptoms, after clinical diagnosis, a DNA test is indicated to confirm the expansion of the GCG-mini repeat in exon 1 of the PABPN1 gene.

## Abbreviations

OPMD: Oculopharyngeal Muscular Dystrophy; ENT: Ear Nose Throat; PEG: Percutaneous Endoscopic Gastrostomy; GCG: Nucleotide Sequences; PABPN1: Gene name: poly(A) binding protein, nuclear 1.

## Consent

Written informed consent was obtained from the patient for publication of this case report and accompanying images. A copy of the written consent is available for review by the Editor-in-Chief of this journal.

## Competing interests

The authors declare that they have no competing interests.

## Authors' contributions

BK as the patient's physician analyzed and interpreted the patient data, ZM performed the genetic screening, BA performed radiological analysis.
